# Clinicopathological Characteristics Predicting Further Recurrence and Survival Following Resection of In-Transit Melanoma Metastases

**DOI:** 10.1245/s10434-022-11997-0

**Published:** 2022-06-30

**Authors:** Anna K. Lawless, David J. Coker, Serigne N. Lo, Tasnia Ahmed, Richard A. Scolyer, Sydney Ch’ng, Omgo E. Nieweg, Kerwin Shannon, Andrew Spillane, Jonathan R. Stretch, John F. Thompson, Robyn P. M. Saw

**Affiliations:** 1 Melanoma Institute Australia, The University of Sydney, Sydney, NSW Australia; 2 Faculty of Medicine and Health, The University of Sydney, Sydney, NSW Australia; 3 Department of Melanoma and Surgical Oncology, Royal Prince Alfred Hospital, Sydney, NSW Australia; 4 Department of Tissue Pathology and Diagnostic Oncology, Royal Prince Alfred Hospital and NSW Health Pathology, Sydney, NSW Australia; 5 Charles Perkins Centre, The University of Sydney, Sydney, NSW Australia; 6 RPA Institute of Academic Surgery, Sydney Local Health District, Sydney, NSW Australia; 7 Department of Breast and Melanoma Surgery, Royal North Shore Hospital, Sydney, NSW Australia

## Abstract

**Background:**

In-transit metastases (ITMs) affect approximately 4% of patients with cutaneous melanoma. This study sought to identify clinical and pathological characteristics that predict further recurrence and survival following resection of ITMs.

**Patients and Methods:**

Patients (*n* = 573) who underwent surgical resection of their first presentation of ITM following previous surgical treatment of an American Joint Committee on Cancer (AJCC) stage I–II melanoma between 1969 and 2017 were identified from an institutional database. Clinicopathological predictors of patterns of recurrence and survival following ITM resection were sought.

**Results:**

The median time of ITM development was 2.4 years after primary melanoma resection. ITMs were most frequently located on the lower limb (51.0%). The most common melanoma subtype associated with ITM development was nodular melanoma (44.1%). After surgical resection of a first ITM, 65.4% of patients experienced recurrent disease. Most recurrences were locoregional (44.7%), with distant metastasis occurring in 23.9% of patients. Lower limb ITMs were more frequently associated with subsequent ITMs [odds ratio (OR) 2.41, *p* = 0.0002], and the lowest risk of distant metastasis (*p* < 0.0001) compared with other primary sites. Primary melanomas and ITM on head and neck, as well as the presence of ulceration, were associated with worse survival.

**Conclusions:**

Recurrence after surgical resection of a first ITM was common. Patterns of recurrence differed according to anatomical site; further ITM recurrences were more likely for lower limb ITMs, which were also associated with longer distant recurrence-free survival. Distant metastasis was more common for ITM on the head and neck, with worse survival.

**Supplementary Information:**

The online version contains supplementary material available at 10.1245/s10434-022-11997-0.

In-transit metastases (ITMs) are cutaneous or subcutaneous metastases located between the primary tumor site and the regional node field,^1–4^ and are thought to occur because of intralymphatic (or possibly angiotropic) tumor spread.^5^ ITMs occur in approximately 4% of all patients with invasive cutaneous melanomas, rising to 11% in patients with thicker primary melanomas.^5–7^

Risk factors for developing ITMs include site of the primary melanoma (limb or trunk), primary tumor characteristics (increased Breslow thickness, higher mitotic rate, lymphovascular invasion, and the presence of ulceration), a positive sentinel lymph node biopsy, and macroscopic regional node involvement.^1, 2, 7, 8^ The latter pathological characteristics are also adverse prognostic indicators for patients with melanoma in general.^5, 7, 9^

Surgical resection remains the standard treatment for operable ITMs. However, there are numerous other locoregional treatment modalities available for ITMs, ranging from topical therapies to isolated limb infusion.^1, 3, 4^ Furthermore, adjuvant systemic treatment is now often recommended for patients with resected stage III melanoma, which includes ITMs.^5^ This includes immunotherapy with checkpoint inhibitors (such as nivolumab, pembrolizumab, or ipilimumab), or targeted therapy in patients with *BRAF*-mutant melanoma (such as combination dabrafenib and trametinib).^10, 11^ Recent trials suggest these drugs are effective treatment after resection of ITMs, however patients with ITMs represent only a very small proportion of the studied cohorts^11, 12^ or were excluded from these trials.^10^ The use of neoadjuvant systemic therapies for stage III melanoma is being assessed in ongoing clinical trials.^13–15^

The use of drug therapies continues to expand in both the adjuvant and neoadjuvant settings. In the absence of other biomarkers, which may become available with time, it is important to identify high- and low-risk subpopulations of patients with ITMs from presently available data to guide management decisions and better stratify patients entering clinical trials. This stratification of patients may spare low-risk patients from adverse events associated with systemic therapy when they are less likely to benefit from treatment, and identify patients at high risk of recurrence, particularly distant recurrence, who stand to gain most from systemic therapy. Hence, this study sought to describe the patterns of recurrence following surgical excision of a first ITM(s) and evaluate the clinical and pathological characteristics that predict recurrence and survival following ITM resection.

## Patients and Methods

### Patient Population

Patients who underwent resection for a first presentation of isolated ITM following previous treatment of a primary AJCC stage I or II cutaneous melanoma at Melanoma Institute Australia (MIA), Sydney, between 1969 and 2017, were identified from a prospectively maintained database. In this database, ITMs are defined as cutaneous or subcutaneous metastases in the same part of the body, separated from the primary lesion by greater than 5 cm, but not in the draining node field. Patients who underwent initial resection of a primary cutaneous melanoma at another center and were referred to MIA for management of their ITM, were included if there was sufficient information on primary tumor pathological characteristics and follow-up.

Patients who first developed other regional or systemic recurrence and subsequently developed ITMs were excluded, as were those with synchronous lymph node or distant metastases at the time of initial ITM diagnosis. Nine patients who received adjuvant immunotherapy after ITM resection were also excluded. Retrieved information included age, gender, location of the primary tumor, melanoma subtype, Breslow thickness, ulceration, stage (AJCC 8th edition), number of ITMs, subsequent recurrence (date and site), date of death or last contact, and cause of death.

### Management

All patients underwent surgery with the intention of achieving complete ITM excision with clear margins, as recommended by current Australian Melanoma Management Guidelines.^8^ ITMs were considered operable where they were not only technically resectable but resection was felt to carry an acceptable morbidity profile compared with other treatment modalities. Sentinel node biopsy was not routinely performed for ITMs at MIA. Throughout the period of the study, clinical follow-up typically occurred every 3–4 months for the initial 2 years, then every 6 months to 5 years. Surveillance imaging and follow-up protocols for patients with ITMs treated at MIA changed over the period of the study. Surveillance ultrasound became more common through the 1990s. Since the early 2000s, patients have routinely undergone full-body computed tomography (CT), and more recently ^18^F-fluorodeoxyglucose positron emission tomography/low dose CT (FDG-PET/CT) scans at the time of ITM diagnosis to assess for the presence of regional and distant metastases, with repeat full-body imaging at least every 12 months.

### Statistical Analysis

Clinical and pathological parameters were analyzed using descriptive statistics, median (and range) for continuous variables and frequency (proportion) for categorical variables. Outcomes included disease-free survival (DFS), overall survival (OS), melanoma-specific survival (MSS), patterns of recurrence (including development of subsequent ITMs), and number of ITMs (count). Survival outcomes were calculated from the date of primary melanoma diagnosis and then from the date of resection of the first ITM. Survival curves were calculated using the Kaplan–Meier method, stratified by Breslow thickness and primary melanoma site. Survival differences between groups were assessed using the log-rank test. Multivariable Cox regression analyses were performed to identify prognostic factors associated with recurrence and survival outcomes in patients with resected ITMs. Associations between baseline factors and disease recurrence (versus no recurrence) were evaluated using univariable logistic regression.

## Results

### Patient Population

Between 1969 and 2017, 1345 patients presented with first ITMs. Of these, 573 patients (43%) underwent surgical resection only and were included in the study. The other 772 ITM patients were found to have either multiple ITMs not suitable for complete resection, or concomitant nodal or distant disease; 174 patients had locoregional disease; 33 had distant metastases; and 565 had both locoregional and distant disease. Over the study period, 44,902 patients with melanoma were entered into the MIA database. Thus, patients presenting with isolated ITMs undergoing surgical resection represented 1.3% of all patients with melanoma who were seen and treated at the institution during the study period.

The median age of the 573 study patients was 68.4 years (range 14.4–95.5 years), and 321 (56.0%) were male. The most common primary melanoma subtypes of the patients presenting with first ITMs were nodular (*n* = 216, 44.1%) and superficial spreading (*n* = 188, 38.4%). The lower limb was the most common site for the primary melanoma (*n* = 292, 51.0%) (Table 1). Median follow-up was 7 years after ITM resection. Median time from resection of the primary melanoma to diagnosis of ITM was 2.4 years (95% CI 2.08–2.75 years) with more than 90% occurring within 10 years of the primary melanoma diagnosis. A single ITM recurrence was the most common, with a range of 1–6 lesions (Table 1).

**Table 1:** Clinical and pathologic characteristics at initial diagnosis

Characteristics	Summary statistics (*n* = 573)
*Clinical characteristics*	
*Gender*	
Female	252 (44.0%)
Male	321 (56.0%)
*Age in years*	
*n*	573
Median (range)	68.4 (14.4, 95.5)
*Age (categorized)*	
≤ 59	149 (26.0%)
60-69	134 (23.4%)
70-77	116 (20.2%)
> 77	174 (30.4%)
*Location of primary*	
Upper extremities	81 (14.1%)
Lower extremities	292 (51.0%)
Trunk	111 (19.4%)
Head and neck	89 (15.5%)
*Pathologic characteristics*	
*Breslow thickness (mm)*	
≤ 1.0	91 (15.9%)
1.1–2.0	158 (27.6%)
2.1–4.0	197 (34.4%)
> 4.0	127 (22.2%)
*Melanoma subtype*	
Acral lentiginous	19 (3.9%)
Lentigo maligna	21 (4.3%)
Nodular	216 (44.1%)
Superficial spreading	188 (38.4%)
Other*	46 (9.4%)
Missing**	83
*Ulceration*	
No	306 (63.0%)
Yes	180 (37.0%)
Missing**	87
*Lymphovascular invasion*	
No	288 (90.3%)
Yes	31 (9.7%)
Missing**	254
*Stage at diagnosis of initial melanoma (AJCC 8th edition)*	
IA	86 (15.0%)
IB	97 (16.9%)
IB/IIA	28 (4.9%)
IIA	134 (23.4%)
IIA/IIB	19 (3.3%)
IIB	132 (23.0%)
IIB/IIC	9 (1.6%)
IIC	63 (11.0%)
Missing	5
*Post-ITM recurrence type*	
No recurrence	198 (34.6%)
Local only + ITM/local	14 (2.4%)
ITM only	94 (16.4%)
Regional only	122 (21.3%)
Regional/local and regional/ITM	8 (1.4%)
Distant only	119 (20.8%)
Distant/ITM and distant/regional	18 (3.1%)
*Number of ITM at first presentation*	
Median (range)	1 (1, 6)
*Number of ITM at first presentation*	
1	552 (96.3%)
2	13 (2.3%)
≥ 3	8 (1.4%)
*Number of further ITMs on follow-up*	
Median (range)	1 (1, 42)
*Number of further ITMs on follow-up*	
0	413 (72.1%)
1	105 (18.3%)
2	25 (4.4%)
≥ 3	30 (5.2%)
*Further ITMs surgically resected*	
Median (range)	2 (1, 29)
*Further ITMs surgically resected*	*n* = 141
1	66 (46.8%)
2	32 (22.7%)
≥ 3	43 (30.5%)

^*^Histologic evaluation revealed 1 case of malignant blue nevus, 6 cases of melanoma in situ, 31 cases of desmoplastic and 9 cases of desmoplastic with neurotropic melanoma

^**^15% of data were missing for melanoma subtype and ulceration, and 44% of data missing for lymphovascular invasion

### Patterns and Timing of Recurrence Following ITM Resection

Following ITM resection, 375 patients (65.4%) experienced melanoma recurrence. Median time to recurrence (of any type) was 16 months from ITM resection (95% CI 13–18 months). The most common pattern of recurrence was locoregional (*n* = 256, 44.7%), including 122 patients (21.3%) with regional nodal disease and 94 patients (16.4%) recurring with further ITMs. There was a trend toward higher regional lymph node recurrence for ITMs resected from the lower limbs compared with those from primary sites on the head and neck, trunk, or upper limbs, although the difference was not statistically significant (HR 1.29, CI 0.78–2.15, *p* = 0.14).

**Fig. 1:**
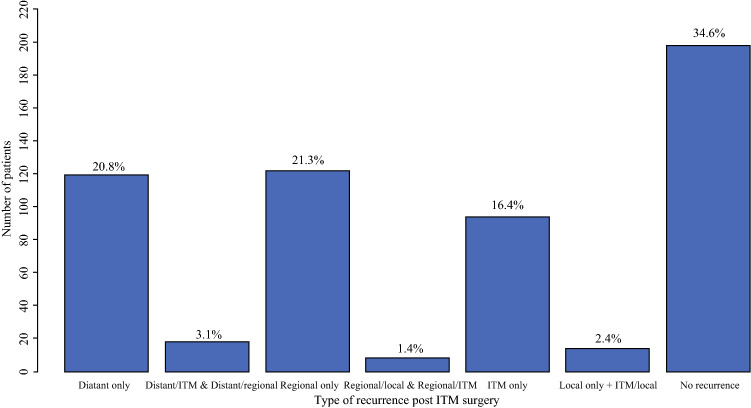
Patterns of recurrence following resection of ITM

Of the entire cohort, 137 patients (23.9%) developed a distant metastasis after ITM resection, either in isolation, or in combination with another recurrence type (Table 1; Fig. 1). Ten-year distant metastasis-free survival (DMFS) rate after ITM resection was 67.5% (95% CI 62.2–73.3%), with median DMFS not reached. Primary melanomas on the upper and lower limbs had significantly lower rates of distant metastasis compared with primary melanomas on trunk or the head and neck (HR 0.51 and HR 0.28, respectively, *p* < 0.0001; Table 2). When patients were stratified by age, younger age (≤ 59 years) was associated with a higher rate of distant metastasis (*p* = 0.0142; Table 2). Breslow thickness and the presence of ulceration or lymphovascular invasion did not predict time to distant recurrence following initial ITM resection (Table 2).

**Table 2:** Univariable and multivariable regression analyses of distant recurrence-free survival from diagnosis of ITM

	Univariable		Multivariable ^†^	
Variable	HR	*p* value	HR	*p* value
*Gender*				
Female	1	0.1722		
Male	1.27 (0.90, 1.79)			
*Age (years)*				
≤ 59	1	0.0346	1	0.0142
60–69	0.92 (0.60, 1.42)		0.86 (0.55, 1.33)	
70–77	0.85 (0.54, 1.36)		0.73 (0.45, 1.17)	
> 77	0.48 (0.29, 0.80)		0.43 (0.25, 0.72)	
*Primary site*				
Head and neck	1	< 0.0001	1	< 0.0001
Lower extremities	0.34 (0.21, 0.53)		0.28 (0.17, 0.45)	
Trunk	0.95 (0.59, 1.52)		0.73 (0.45, 1.21)	
Upper extremities	0.63 (0.36, 1.09)		0.51 (0.29, 0.90)	
*Breslow thickness*				
≤ 1.0	1	0.6336		
> 1–2.0	1.32 (0.77, 2.28)			
> 2.0–4.0	1.13 (0.66, 1.94)			
> 4.0	1.37 (0.78, 2.40)			
*Histology*				
Acral lentiginous	1	0.4749		
Lentigo maligna melanoma	2.79 (0.56, 13.84)			
Nodular melanoma	2.50 (0.61, 10.26)			
Superficial spreading	1.94 (0.47, 8.04)			
Other	2.76 (0.62, 12.32)			
*Ulceration*				
No	1	0.6846		
Yes	1.08 (0.74, 1.58)			
*Lymphovascular invasion*				
No	1	0.3965		
Yes	0.67 (0.27, 1.67)			

^**†**^The multivariate model was determined using the backward elimination technique with all the significant (*p*-value ≤ 0.20) variables from the univariate analysis

### Further ITMs After ITM Resection

ITMs on the lower extremities were strongly associated with further ITMs after initial surgical resection (OR 2.41, 95% CI 1.35–4.31, *p* = 0.0002; Supplementary Table 2); this was not the case for other primary sites. Age, gender, Breslow thickness, melanoma subtype, ulceration, and lymphovascular invasion were not associated with further recurrence of ITMs after surgical resection. For patients who recurred with further ITMs after initial ITM resection, the median number of further (separate) ITM excisions ranged from 1 to 29 over the study period (Table 1). Most of the further ITMs were within 2 years of initial ITM resection.

### Patients Without Disease Recurrence After ITM Resection

Of the 573 patients who underwent surgical resection of ITMs 198 patients (34.6%) did not recur. These patients were older than those who experienced disease recurrence (median 72.5 versus 67.0 years, *p* = 0.0001). There was no difference between groups regarding gender, location of primary, Breslow thickness, melanoma subtype, presence of ulceration, AJCC stage at diagnosis, or number of ITMs at presentation.

### Survival Following ITM Resection

**Fig. 2:**
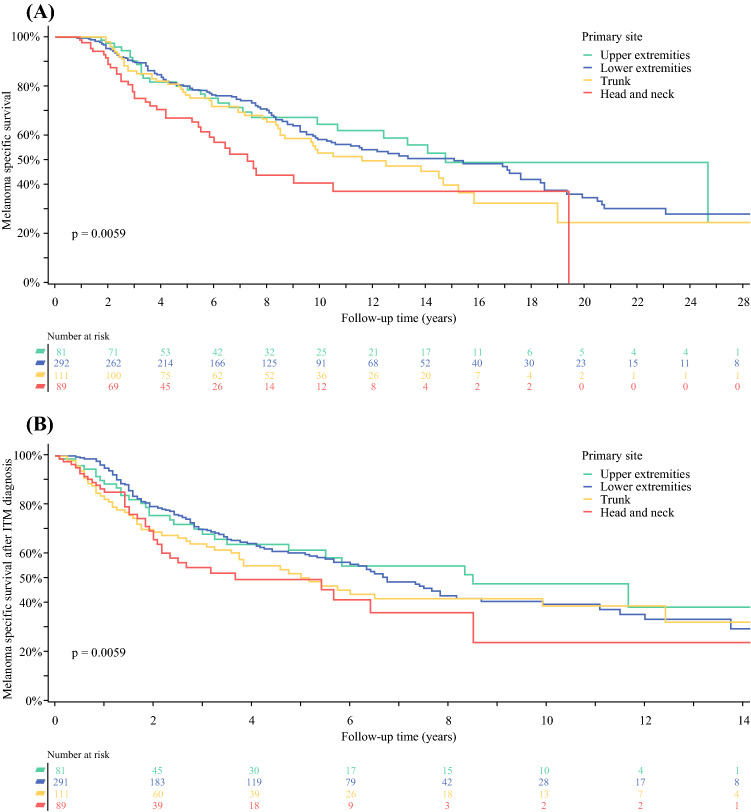
Melanoma-specific survival from time of primary diagnosis (A) and ITM diagnosis (B) stratified by primary site

From the time of primary melanoma diagnosis, the median melanoma-specific survival (MSS) and overall survival (OS), was 13 and 9.1 years, respectively. From the time of ITM resection, MSS was 6.4 years and OS was 4.1 years. The presence of ulceration in the primary melanoma was associated with worse MSS from both time of primary melanoma diagnosis (HR 1.49, *p* = 0.0099) and ITM resection (HR 1.52, *p* = 0.0038). From the time of primary diagnosis, melanomas on the head and neck, and those with increased Breslow thickness were associated with worse MSS (Table 3; Fig. 2). Primary site on the head and neck showed a trend toward worse MSS when calculated from time of ITM resection (*p* = 0.09; Table 3).

**Table 3:** Univariable and multivariable regression analyses of melanoma-specific survival

	From primary melanoma diagnosis				From ITM diagnosis	
	Univariable		Multivariable (†)		Univariable	
Variable	HR (95% CI)	*p* value	HR (95% CI)	*p* value	HR	*p* value
*Gender*						
Female	1	0.0277			1	0.2163
Male	1.34 (1.03, 1.74)				1.18 (0.91, 1.52)	
*Age (years)*						
≤ 59	1	0.0023			1	0.2531
60–69	1.06 (0.75, 1.51)				0.86 (0.61, 1.22)	
70–77	1.16 (0.79, 1.70)				0.80 (0.55, 1.17)	
> 77	1.87 (1.31, 2.65)				1.14 (0.81, 1.60)	
*Primary site*						
Head and neck	1	0.0072	1	0.008	1	0.0929
Lower extremities	0.54 (0.37, 0.79)		0.50 (0.33, 0.75)		0.65 (0.45, 0.94)	
Trunk	0.66 (0.43, 1.01)		0.59 (0.36, 0.95)		0.79 (0.52, 1.22)	
Upper extremities	0.49 (0.30, 0.80)		0.48 (0.28, 0.83)		0.61 (0.37, 1.00)	
*Breslow thickness*						
≤ 1.0	1	0.0018	1	0.0179	1	0.4666
> 1–2.0	1.23 (0.81, 1.86)		1.54 (0.90, 2.65)		1.22 (0.80, 1.84)	
> 2.0–4.0	1.72 (1.15, 2.58)		2.11 (1.24, 3.60)		1.34 (0.90, 2.00)	
> 4.0	2.07 (1.34, 3.18)		2.20 (1.24, 3.90)		1.37 (0.89, 2.10)	
*Histology*						
Acral lentiginous	1	0.3962			1	0.531
Lentigo maligna melanoma	0.43 (0.13, 1.42)				0.40 (0.12, 1.34)	
Nodular melanoma	1.04 (0.51, 2.15)				0.90 (0.44, 1.86)	
Superficial spreading	0.89 (0.43, 1.84)				0.83 (0.40, 1.71)	
Other	0.81 (0.34, 1.96)				0.73 (0.30, 1.75)	
*Ulceration*						
No	1	< 0.0001	1	0.0099	1	0.0038
Yes	1.80 (1.35, 2.38)		1.49 (1.10, 2.01)		1.52 (1.14, 2.01)	
*Lymphovascular invasion*						
No	1	0.3161			1	0.8603
Yes	1.38 (0.74, 2.57)				1.06 (0.57, 1.97)

Regarding OS after ITM resection, age > 77 years (HR 1.88, *p* = 0.0005), presence of ulceration in the primary melanoma (HR 1.38, *p* = 0.0079), and ITM on the head or neck (*p* = 0.0179) were associated with significantly worse OS (Supplementary Table 1).

## Discussion

Resection with clear margins remains the gold standard for management of operable melanoma ITMs. However, recurrence rates are high, and the timing, risk factors, and patterns of recurrence have not been well described in literature. Our study provides important information on favorable and unfavorable prognostic factors after ITM resection. This may assist in tailoring management, which today includes not only the options of a range of local and regional therapies,^1, 3, 4^ but also the use of adjuvant and neoadjuvant systemic therapies. Our findings may also assist in the design of further trials involving patients with ITMs.

### Melanoma Subtypes Associated with ITMs

Our finding that resected ITMs were more likely to occur after a nodular primary melanoma (44.1%) has not been reported previously. This suggests that nodular melanoma may have an inherently greater biological propensity for intralymphatic (or possibly angiotropic) tumor spread, which warrants further investigation.

### Patterns and Timing of Recurrence After Resected ITM

We report a recurrence rate of 65.4% after resection of an initial ITM, in keeping with previous retrospective series suggesting recurrence rates (local, regional, or distant) of 63–72%.^16, 17^ Nearly half of the cohort experienced locoregional recurrence (44.7%), with similar rates of isolated regional lymph node and ITM recurrences. Less than a quarter developed distant metastases over the extended follow-up period. This should be borne in mind when considering management options in the contemporary era of systemic therapy for metastatic melanoma.

### Risk Factors for Survival After ITM Resection

ITMs from primary melanomas on the head and neck were associated with the highest rates of distant metastasis and a trend to worse MSS from the time of ITM diagnosis. There is inherent difficulty in differentiating in-transit disease on the head and neck from systemic dermal metastases in the context of midline head and neck lesions, which may contribute to this finding, though it is considered unlikely to be a dominant confounder. Increased Breslow thickness was associated with worse MSS from both time of primary melanoma and from time of ITM despite not having a clear association with development of distant metastases.

Younger age was associated with high rates of distant metastasis after ITM resection but was only associated with worse MSS from time of primary melanoma, not from time of ITM resection. This finding suggests that younger patients should be considered for adjuvant systemic therapy after ITM resection at a lower threshold than older patients because they are at higher risk of distant metastasis, and therefore stand to benefit more from systemic therapy.

### Patterns and Timing of Recurrence After ITM Resection

Following ITM resection, 16.4% of patients recurred with further ITMs, approximately four times greater than the rate of ITMs expected following excision of a primary cutaneous melanoma. This suggests an inherent biological propensity of the melanoma in these patients to spread and lodge intralymphatically. Patients with first ITMs on the lower limb were more than twice as likely to recur with further ITMs, compared with ITMs located elsewhere on the body. This may reflect increased intralymphatic hydrostatic pressure in the lower limb or possibly the greater surface area of the lower extremity upon which to recur.

The high rates of both nodal and distant recurrence after ITM resection support the use of regular imaging surveillance in this patient cohort. In particular combining ultrasound, owing to its high sensitivity and specificity in diagnosing nodal disease,^18^ with PET/CT to assess both nodal and distant recurrences.^19^ Ultimately, the disparate patterns of recurrence following resection of ITMs suggest the need for a multimodal approach to surveillance, incorporating clinical examination, ultrasound, and cross-sectional imaging.

### Use of Adjuvant Systemic Therapy After ITM Resection

In attempting to stratify high risk and low risk populations with respect to patterns of recurrence and survival, our results suggest that early consideration of systemic therapy in patients with head and neck ITMs may be advisable, given their increased risk of distant recurrence and worse survival. Similarly, ITMs in younger patients were associated with increased rates of subsequent distant recurrence, which appeared to impact MSS, suggesting that these patients stand to gain most from systemic therapy.

In contrast, patients with resected ITMs on the limbs had lower rates of distant metastasis, with improved MSS, compared with those with ITMs on the trunk or head and neck. This would appear to justify more aggressive local management of limb ITMs, particularly surgical resection when recurrences are amenable to operation, reserving systemic therapies for subsequent non-operable regional relapse or distant metastasis. Such an approach seeks to maximize potential benefit from systemic therapies in the context of ITMs while minimizing potential adverse effects.

### Limitations

This study is strengthened by the large cohort size, extended follow-up time, and prospective nature of the database from which information was obtained. However, inherent limitations in the study design as an observational, single-institutional study remain. Due to the long study period (1969–2017), both the definition of operability and surveillance regimens undoubtedly changed over the course of the study. The concept of operable ITMs is not fixed, and is likely to have been a trend for less extensive surgery when other treatment modalities became available. Unfortunately, the MIA database lacks sufficient detail to reflect precisely the decision-making process with respect to operable versus nonoperable lesions in this cohort over the study period.

Surveillance practices also changed over the study period. The use of ultrasound increased through the 1990s, with improvements in technical sophistication and clinical expertise. Similarly, FDG-PET became incorporated into routine use a decade later. These changes likely improved staging and post-treatment surveillance, which would be expected to have improved outcomes by excluding those with occult metastatic disease from upfront surgery and allowing earlier detection and potential salvage at the time of relapse.

Despite the inherent limitations outlined above, this study documents the natural history of melanoma after resection of ITMs prior to the era in which potentially effective systemic therapies have become widely available to treat metastatic melanoma.

## Conclusions

Following surgical resection of isolated ITMs, after previous wide excision of a primary stage I or II melanoma, 65.4% of patients experienced disease recurrence. Patterns of recurrence differed by anatomical sites lower limb ITMs more likely to recur with further ITMs, with but much less likely with distant metastasis. Head and neck ITMs and younger age were associated with increased rates of distant metastasis. This suggests that operable ITMs on the lower limb should be resected up front, and adjuvant drug therapy possibly held in reserve until further recurrence. However, operable ITMs in younger patients and those on the head and neck should be considered for adjuvant and possibly neoadjuvant drug therapy. Our data are useful for stratifying higher risk and lower risk subpopulations of patients with melanoma who develop ITMs to allow a more tailored approach to the institution of locoregional or systemic therapies within a multidisciplinary treatment environment.

## Supplementary Information

Below is the link to the electronic supplementary material.

Supplementary file1 (DOCX 26 kb)
