# Supplementary material for: Clinicopathological Characteristics Predicting Further Recurrence and Survival Following Resection of In-Transit Melanoma Metastases
Source: Ann Surg Oncol. 2022 Jun 30;29(11):7019–28. doi: 10.1245/s10434-022-11997-0 (PMC9492704; doi:10.1245/s10434-022-11997-0)
Supplement: Supplementary file 1 — Supplementary file1 (DOCX 26 kb) [file 10434_2022_11997_MOESM1_ESM.docx]

**Supplementary Tables and Figures**

**List of Supplementary Tables**

Table 1. Overall survival from primary diagnosis and ITM diagnosis

Table 2. Univariate logistic regression of further recurrence of ITM

**Table 1. Univariable and Multivariable Regression of Overall Survival**

|  | **From Time of Primary Melanoma** | | | | **From Time of ITM Diagnosis** | | | |
| --- | --- | --- | --- | --- | --- | --- | --- | --- |
|  | **Univariable** | | **Multivariable (†)** | | **Univariable** | | **Multivariable** | |
| **Variable** | **HR** | **P-value** | **HR** | **P-value** | **HR** | **P-value** | **HR** | **P-value** |
| **Gender** |  |  |  |  |  |  |  |  |
| Female | 1 | **0.0065** | 1 | **0.0233** | 1 | 0.1498 |  |  |
| Male | 1.35 (1.09, 1.67) |  | 1.32 (1.04, 1.67) |  | 1.17 (0.94, 1.45) |  |  |  |
| **Age(years)** |  |  |  |  |  |  |  |  |
| ≤59 | 1 | **<.0001** | 1 | **<.0001** | 1 | **<.0001** | 1 | **0.0005** |
| 60-69 | 1.41 (1.03, 1.93) |  | 1.28 (0.88, 1.85) |  | 1.11 (0.81, 1.52) |  | 1.15 (0.80, 1.65) |  |
| 70-77 | 2.14 (1.55, 2.95) |  | 1.94 (1.35, 2.79) |  | 1.40 (1.02, 1.92) |  | 1.40 (0.99, 1.99) |  |
| >77 | 3.37 (2.48, 4.58) |  | 2.69 (1.90, 3.82) |  | 1.91 (1.43, 2.56) |  | 1.88 (1.36, 2.60) |  |
| **Primary site** |  |  |  |  |  |  |  |  |
| Head and Neck | 1 | **0.0002** |  |  | 1 | **0.0179** |  |  |
| Lower extremities | 0.51 (0.37, 0.69) |  |  |  | 0.61 (0.45, 0.83) |  |  |  |
| Trunk | 0.57 (0.40, 0.82) |  |  |  | 0.67 (0.47, 0.96) |  |  |  |
| Upper extremities | 0.51 (0.35, 0.76) |  |  |  | 0.65 (0.44, 0.96) |  |  |  |
| **Breslow thickness** |  |  |  |  |  |  |  |  |
| ≤1.0 | 1 | **<.0001** | 1 | **0.017** | 1 | 0.0878 |  |  |
| >1-2.0 | 1.27 (0.90, 1.81) |  | 1.33 (0.85, 2.10) |  | 1.27 (0.89, 1.80) |  |  |  |
| >2.0-4.0 | 1.90 (1.36, 2.67) |  | 1.80 (1.15, 2.83) |  | 1.48 (1.05, 2.07) |  |  |  |
| >4.0 | 2.31 (1.61, 3.31) |  | 1.92 (1.18, 3.12) |  | 1.51 (1.05, 2.16) |  |  |  |
| **Histology** |  |  |  |  |  |  |  |  |
| Acral Lentiginous | 1 | 0.4896 |  |  | 1 | 0.503 |  |  |
| Lentigo Maligna Melanoma | 0.61 (0.27, 1.39) |  |  |  | 0.56 (0.25, 1.25) |  |  |  |
| Nodular Melanoma | 0.84 (0.48, 1.46) |  |  |  | 0.69 (0.40, 1.20) |  |  |  |
| Superficial Spreading | 0.70 (0.40, 1.23) |  |  |  | 0.62 (0.36, 1.09) |  |  |  |
| Other | 0.81 (0.42, 1.58) |  |  |  | 0.72 (0.37, 1.39) |  |  |  |
| **Ulceration** |  |  |  |  |  |  |  |  |
| No | 1 | **<.0001** | 1 | **0.0067** | 1 | **0.0062** | 1 | **0.0079** |
| Yes | 1.61 (1.27, 2.04) |  | 1.42 (1.10, 1.83) |  | 1.39 (1.10, 1.76) |  | 1.38 (1.09, 1.74) |  |
| **Lympho-vascular invasion** |  |  |  |  |  |  |  |  |
| No | 1 | 0.0814 |  |  | 1 | 0.6446 |  |  |
| Yes | 1.55 (0.95, 2.52) |  |  |  | 1.12 (0.69, 1.83) |  |  |  |

**Table 2. Univariate Logistic Regression of Further Recurrence of ITM**

|  | **Univariable** | |
| --- | --- | --- |
| **Covariates** | **OR** | **P-value** |
| **Gender** |  |  |
| Female | 1 | 0.3311 |
| Male | 0.83 (0.58, 1.20) |  |
| **Age(years)** |  |  |
| <=59 | 1 | 0.7849 |
| 60-69 | 0.98 (0.59, 1.64) |  |
| 70-77 | 0.84 (0.49, 1.45) |  |
| >77 | 0.81 (0.50, 1.31) |  |
| **Primary site** |  |  |
| Head and Neck | 1 | **0.0002** |
| Lower extremities | 2.41 (1.35, 4.31) |  |
| Trunk | 1.11 (0.55, 2.23) |  |
| Upper extremities | 0.96 (0.45, 2.08) |  |
| **Breslow thickness** |  |  |
| <=1.0 | 1 | 0.6052 |
| 1.1-2.0 | 0.95 (0.53, 1.70) |  |
| 2.1-4.0 | 1.11 (0.63, 1.94) |  |
| >4.0 | 1.33 (0.73, 2.42) |  |
| **Histology** |  |  |
| Acral lentiginous | 1 | 0.3480 |
| Lentigo maligna | 0.35 (0.09, 1.34) |  |
| Nodular | 0.40 (0.15, 1.03) |  |
| Superficial spreading | 0.47 (0.18, 1.22) |  |
| Other | 0.35 (0.11, 1.08) |  |
| **Ulceration** |  |  |
| No | 1 | 0.5822 |
| Yes | 1.12 (0.75, 1.69) |  |
| **Lympho-vascular invasion** |  |  |
| No | 1 | 0.8769 |
| Yes | 1.06 (0.48, 2.35) |  |

********Only ulceration was selected from backward elimination technique. Hence no multivariable model has been determine*
